# Juvenile Spontaneous Mediastinal Hemorrhage with Suspect Trauma

**DOI:** 10.3390/ani16203223

**Published:** 2026-10-14

**Authors:** Abigail G. Uhrinek, Katharine E. Peper, Adam W. Stern, Federico R. Vilaplana Grosso

**Affiliations:** College of Veterinary Medicine, University of Florida, Gainesville, FL 32608, USA; peper.kate@ufl.edu (K.E.P.); adamstern@ufl.edu (A.W.S.); fvilaplanagrosso@ufl.edu (F.R.V.G.)

**Keywords:** mediastinum, idiopathic hemorrhage, postmortem computed tomography, sentinel clot sign, canine

## Abstract

A 6-month-old intact female American Pitbull (*Canis lupus familiaris*) underwent forensic evaluation for suspected blunt force trauma. Postmortem computed tomography (PMCT) identified pleural hyperattenuating soft tissue attenuating material, consistent with clotted blood, pleural effusion, a large thymus, and moderate to severe cranial mediastinal expansion with mixed fluid and lower-attenuation, soft tissue attenuating material. Histology confirmed thymic hemorrhage, while autopsy identified hemothorax, pleural blood clots, and hemomediastinum, excluding trauma, most consistent with spontaneous idiopathic thymic hemorrhage. This case highlights the utility of identifying hyperattenuating clot formation on PMCT, like the sentinel clot sign, for localizing antemortem hemorrhage and improving antemortem-postmortem correlation in forensic investigations.

## Simple Summary

A 6-month-old female American Pitbull was evaluated given concern for blunt force trauma causing death. Postmortem computed tomography revealed blood in the chest, in the area between the heart and lungs as well as between the lungs and body wall. Clotted blood was found between the right lungs and the surrounding body wall. Examination after death confirmed these findings. The thymus, an organ in the chest of young animals, was large and contained areas of bleeding. No definitive evidence of trauma was identified. The findings were most consistent with a spontaneous thymic bleed, with no identified cause. This case demonstrates that the use of computed tomography after death is useful in locating the source of internal bleeding, providing useful information regarding cause of death in veterinary forensic investigations.

## 1. Introduction

The “sentinel clot sign” on computed tomography involves the presence of a high-density clot with concurrently high hemoglobin content and high attenuation near the source of a bleed [1]. Using Hounsfield Units (HU), the sentinel clot sign can be used to identify a bleeding organ [1]. The attenuation range for a sentinel clot sign has been defined as 56–70 HU, with the mean HU being 56, compared to concurrent hemoabdomen at 20–45 HU [1]. Like human medicine [2], veterinary medicine has used the sentinel clot sign to accurately localize the source of hemoabdomen in patients, subsequently confirmed at autopsy [1,3].

Hemomediastinum, a relatively uncommon condition in dogs [4], has many causes, including trauma, anticoagulant rodenticide toxicity [5,6,7], thymic hemorrhage [6,8,9], and neoplastic conditions [10,11]. Idiopathic thymic hemorrhage is an uncommon cause of hemomediastinum in young dogs (<2 years old) with no breed or sex predilection [8]. Approximately seventy cases had been documented in dogs prior to 1994 [8,9,12,13,14], with few additional cases documented since, typically in animals having no history of trauma, coagulopathy, or neoplasia [15]. Due to the fatal nature of the disease, these cases were documented and diagnosed at necropsy [8]. The clinical presentation of idiopathic thymic hemorrhage can include lethargy, pyrexia, dyspnea, and pain on palpation of the cranial thorax [8,15]. Although the pathogenesis remains poorly understood, one proposed mechanism is hemorrhage secondary to increased fragility in the thymic vascular beds during thymic regression, which occurs at 6–12 months of age in dogs [9,16]. Previous reports describe radiographic findings to include a cranial mediastinal mass [8], while computed tomography has demonstrated thymic involvement, pleural effusion, and mediastinal widening [15]. Prior postmortem examinations have identified generalized tissue pallor, thymic hemorrhage, cranial hemomediastinum and hemothorax, atelectatic lungs, and alveolar hemorrhage [8]. More recently, the mortality rate associated with conservative management is 30% [15], whereas surgical thymectomy can be curative in affected dogs [17]. 

Because spontaneous idiopathic thymic hemorrhage may closely resemble traumatic hemorrhage clinically and at postmortem examination, distinguishing between the two can be challenging. Previous reports have stated that even minor trauma may contribute to fatal thymic hemorrhage [9]. As such, excluding other differentials and accurately recognizing this condition is vital during veterinary forensic investigations to avoid misdiagnoses.

The aim of this report is to describe the postmortem computed tomography, autopsy, and the histopathologic findings of spontaneous thymic hemorrhage in a young dog. This report highlights the utility of postmortem computed tomography with corresponding autopsy for the identification and localization of internal hemorrhage during veterinary forensic investigations. 

## 2. Materials and Methods

### 2.1. Clinical History and Presentation

A juvenile (approximately 6-month-old), intact female, American Pitbull dog (*Canis lupus familiaris*) was brought to an emergency clinic and was unresponsive upon arrival. During triage, blood was noted coming from the mouth, along with multiple loose deciduous teeth, pale gums, and rapidly declining vital signs. A PCV of 15% was identified, and during the examination, the animal began to cough up large amounts of blood, which led to the decision for humane euthanasia. Prior to the visit, a neighbor had expressed concern for blunt force trauma and that the animal was being intentionally harmed, given that they witnessed teeth falling from the animal’s mouth. The body was submitted to a veterinary forensic sciences laboratory for postmortem computed tomography (PMCT), which was performed three days after death, and was subsequently frozen until a forensic autopsy was performed 13 days after death. Prior to the PMCT, the body was stored for three days in a freezer at −20 °C and was thawed in plastic bags in a warm water bath.

### 2.2. Postmortem Computed Tomography (PMCT) 

Helical computed tomography (University of Florida College of Veterinary Medicine, Gainesville, FL, USA; Aquilion Prime, Toshiba Medical Systems, Tustin, CA, USA; 160-slice MDCT, slice thickness from 0.625 to 5 mm based on reconstruction and patient size, pitch 0.8 mm, tube rotation time 0.5 s, matrix size 512 × 512, 120 kVp tube voltage, 100 mAs tube current) data were obtained three days postmortem for further evaluation given the suspicion of blunt force trauma. Images were obtained with the patient placed in right lateral recumbency, in soft tissue, bone, and lung algorithms with 3 mm soft tissue window and 2 mm lung window slice thickness, and reformatted in transverse, dorsal, and sagittal planes. A veterinary diagnostic imaging resident and a board-certified ACVR veterinary radiologist interpreted the images.

### 2.3. Autopsy 

The autopsy was 13 days postmortem and was conducted by a board-certified veterinary pathologist. A gross postmortem examination was conducted, including evaluation of the external body surface and the internal (thoracic, abdominal, and cranial) cavities. Tissue samples were collected from the thymus, lung, liver, kidney, spleen, small intestine, heart, brain, skeletal muscle, skin, adrenal, thyroid, trachea, esophagus, stomach, large intestine, and omentum during the autopsy and were fixed in 10% neutral buffered formalin, routinely processed in paraffin, sectioned, and stained with hematoxylin and eosin (H&E) for histologic evaluation. Tissues were examined by light microscopy. 

### 2.4. Generative AI

Mendeley Reference Manager, version 2.150.0, was used for reference management, citation organization, and bibliography formatting during manuscript preparation.

## 3. Results

### 3.1. PMCT Findings

**Figure 1:**
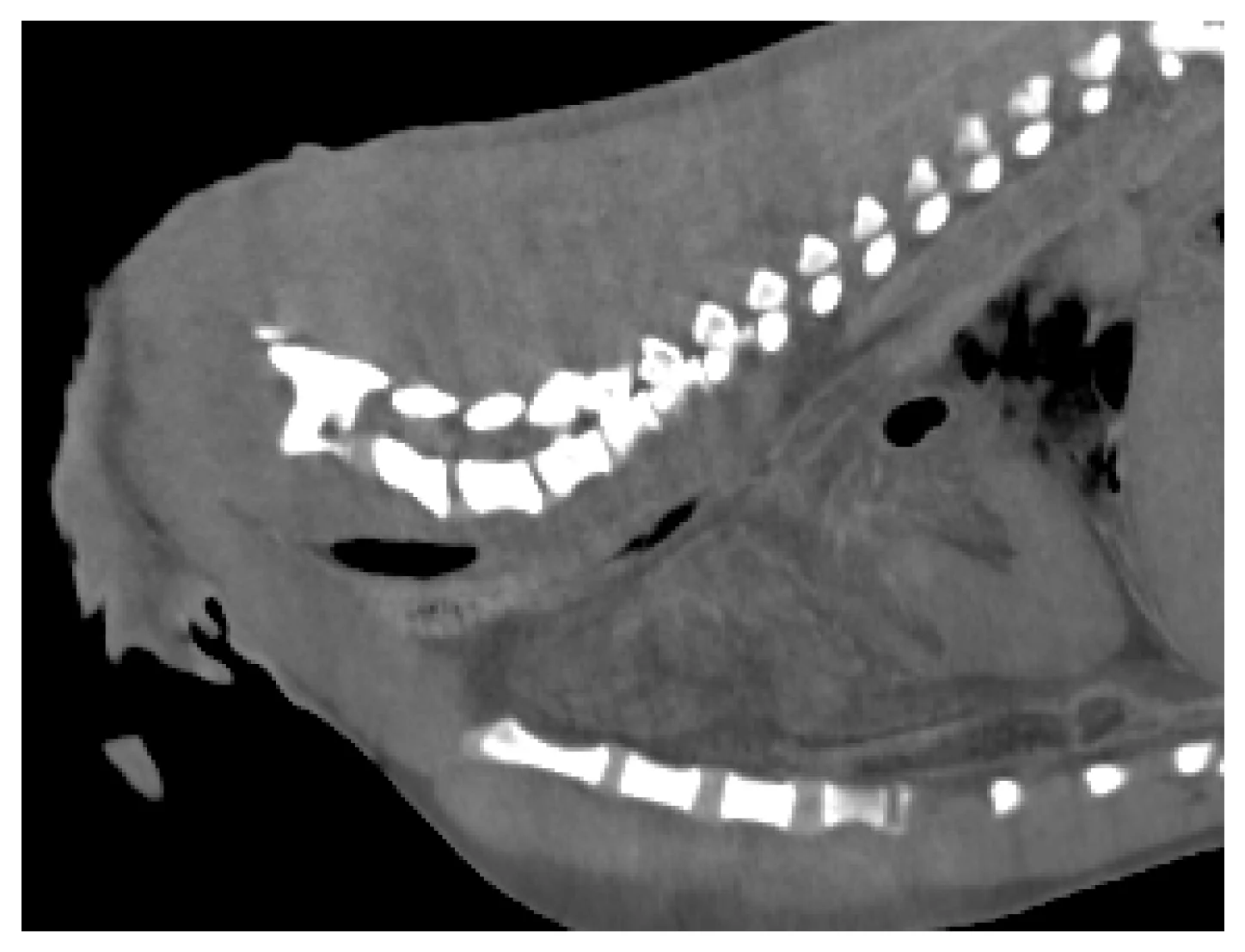
Sagittal postmortem computed tomography (PMCT) image, captured postmortem, highlighting the thoracic cavity in a soft tissue window (window width: 400, level: 40 HU) of a 6-month-old, female intact American Pitbull. The cranial mediastinum is expanded by heterogeneous, mixed fluid-to-soft-tissue attenuating material, occupying the entirety of the cranial mediastinum, compatible with the identified cranial mediastinal hemorrhage.

**Figure 2:**
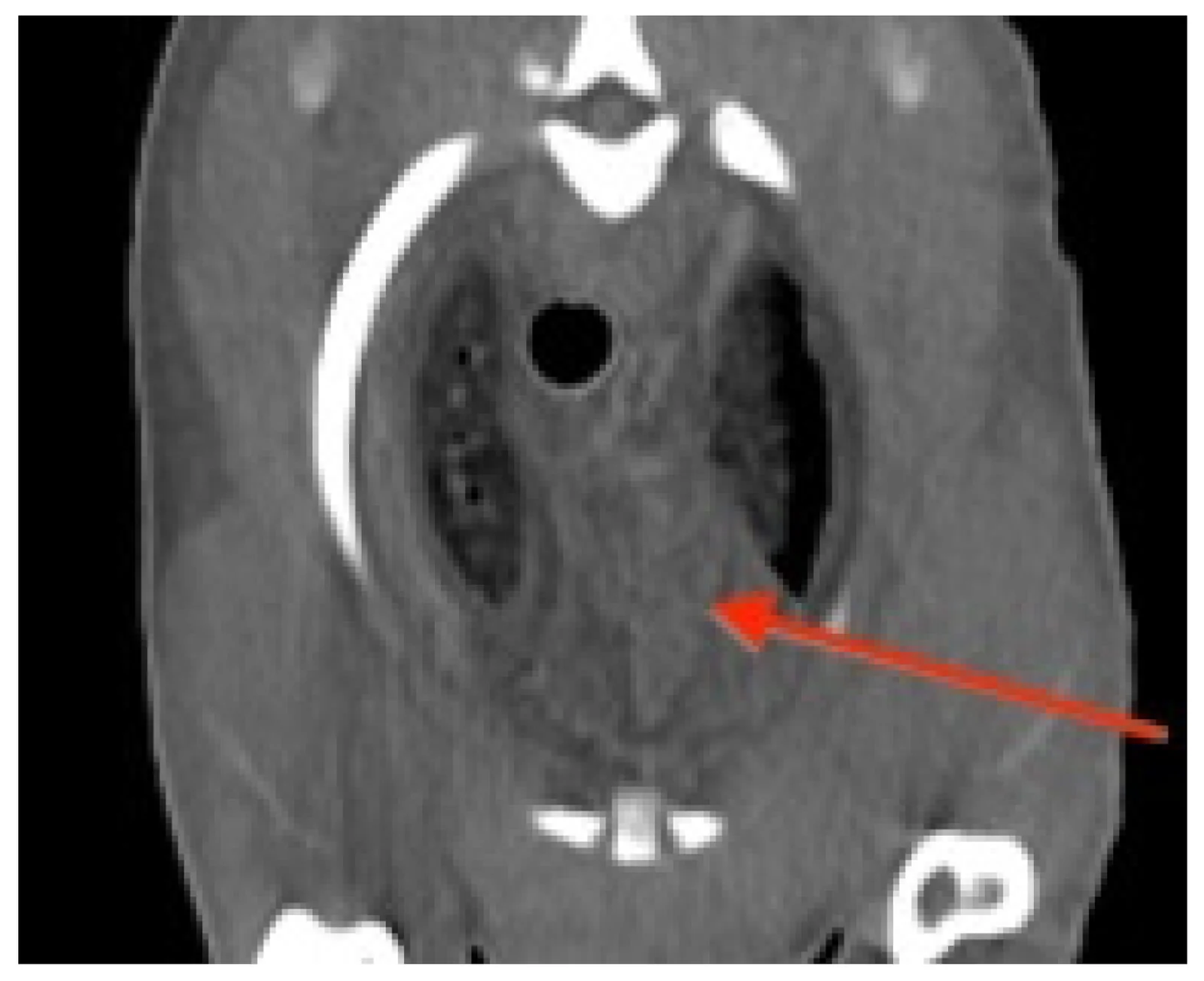
Transverse PMCT image captured postmortem of a 6-month-old, female intact American Pitbull, demonstrating the thorax at the level of the fourth ribs in a soft tissue window (window width: 400, level: 40 HU) with the patient positioned in right lateral recumbency. The cranial mediastinum is expanded by heterogeneous, mixed fluid-to-soft-tissue attenuating material, occupying the entirety of the cranial mediastinum, compatible with the identified cranial mediastinal hemorrhage. There is diffuse expansion of the thymic parenchyma (red arrow).

**Figure 3:**
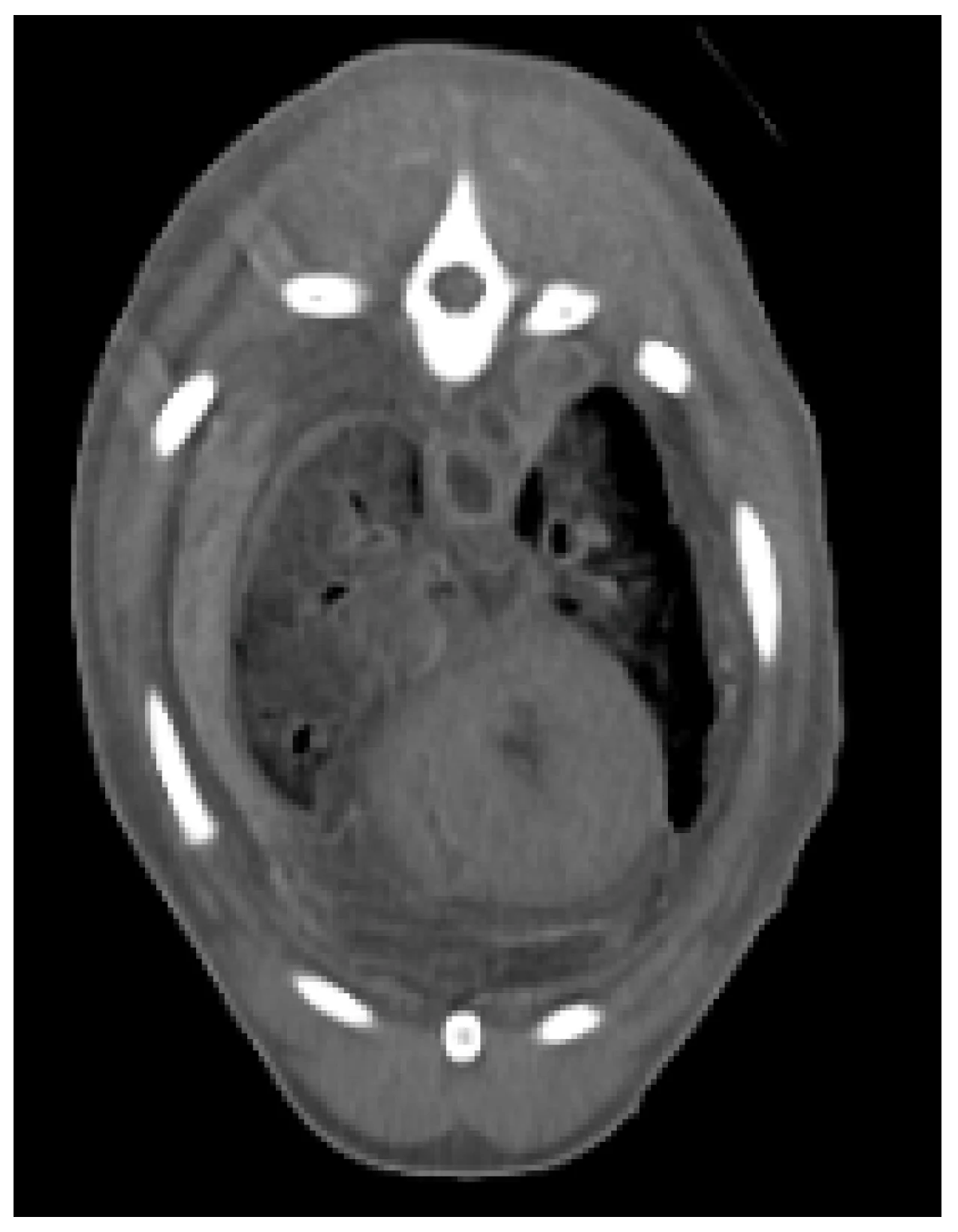
Transverse PMCT image captured postmortem of a 6-month-old, female intact American Pitbull, demonstrating the thorax at the level of the eighth thoracic vertebra in a soft tissue window (window width: 400, level: 40 HU) with the patient positioned in right lateral recumbency. The right pleural space is expanded by a moderate volume of relatively hyperattenuating soft tissue attenuating material (up to 57 HU), resulting in retraction of the lung lobes, consistent with the identified pleural blood clots.

Major PMCT findings included moderate to severe fluid-to-soft-tissue attenuating material in the cranial mediastinum (Figure 1 and Figure 2), with diffuse expansion of the thymic parenchyma (Figure 2). Moderate to severe accumulation of soft tissue, hyperattenuating material (up to 57 HU) with concurrent lung lobe retraction was present in the pleural space bilaterally, more severe on the right side (Figure 3). Additionally, the axial and appendicular skeleton was normal, without fracture, abrasions, or penetrating injury identified. Imaging findings were most consistent with antemortem hemomediastinum and hemothorax, with associated pleural clot formation.

### 3.2. Autopsy Findings

**Figure 4:**
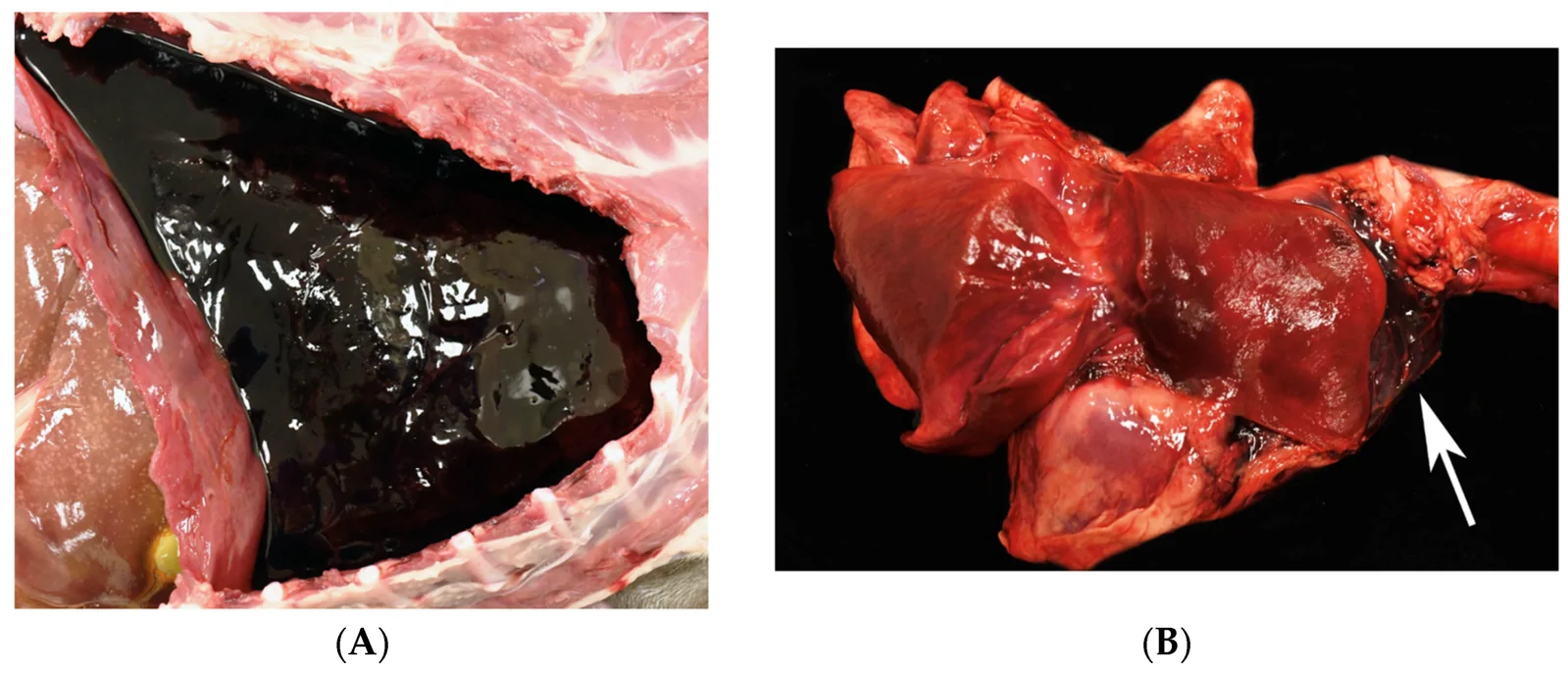
Gross postmortem images obtained during the autopsy of a 6-month-old intact female American Pitbull. (**A**) Thoracic cavity filled with blood and blood clots. (**B**) Heart and lungs, with the white arrow indicating hemorrhagic expansion of the cranial mediastinum.

**Figure 5:**
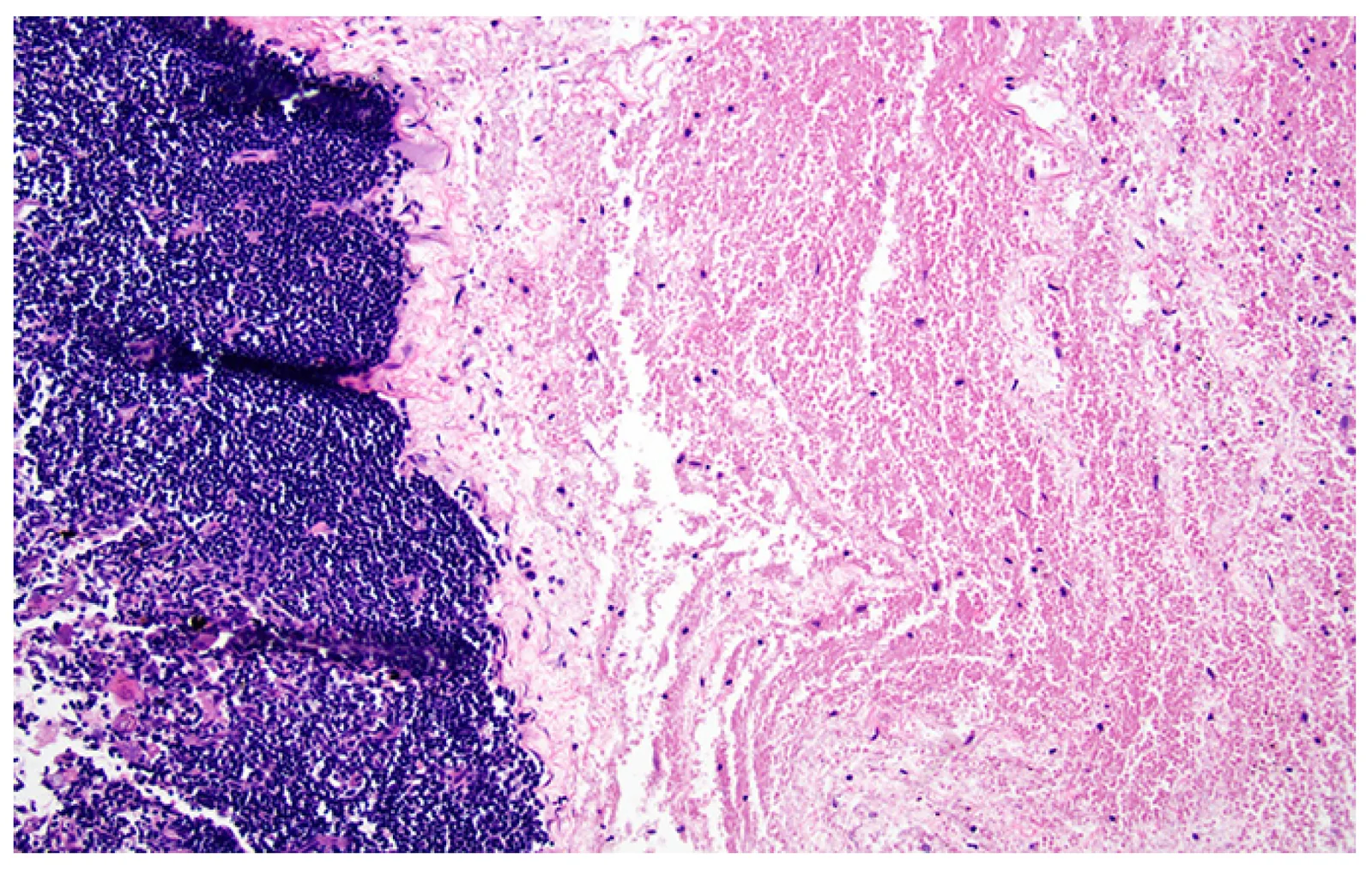
Photomicrograph of the thymus and mediastinum demonstrating expansion of the mediastinum by extravasated erythrocytes (hemorrhage). Hematoxylin and eosin, 200×.

The dog was in ideal nutritional condition with adequate subcutaneous fat and muscling. There was no evidence of ectoparasites, cutaneous punctures, lacerations, or abrasions. A small amount of dried red material was present on the left side of the lips, and body-wide the musculature appeared paler than expected; severe anemia was documented at autopsy. Examination of the thoracic cavity revealed approximately 250 mL of dark red, thick fluid and predominantly a right-sided accumulation of dark red gelatinous material that was free-floating or loosely adhered to the lungs (Figure 4A), along with moderate expansion of the cranial mediastinum in the region of the thymus containing similar dark red fluid and gelatinous material (Figure 4B). No rib, skull, or long bone fractures were identified. Red-tinged froth was present within the trachea, and the lungs were moderately heavy, wet, soft, and mildly collapsed, with pink left lung lobes and red-brown right lung lobes. No other significant changes were observed during the autopsy. All loose teeth corresponded to deciduous dentition. Histology of the thymic tissue revealed that the thymic cortex and medulla, along with the supporting mediastinal tissue, were markedly expanded by extravasated erythrocytes (Figure 5). There was no significant inflammatory response, hemosiderophage accumulation, or hematin deposition within the thymus or mediastinum. No other significant microscopic abnormalities were observed in the remaining tissues examined. Collectively, these findings were consistent with severe hemothorax and intrathoracic blood clots, with moderate mediastinal/thymic hemorrhage. Using an estimated blood volume of 85 mL/kg, the 15.2 kg dog had an estimated total circulating blood volume of 1292 mL. Using the combination of the approximately 250 mL of intrathoracic blood and three blood clots weighing 18 g, 116.2 g, and 51.2 g (assuming 1 g of clot mass approximates 1 mL of blood), which represents an estimated total blood loss of 435.4 mL and is equivalent to approximately 33.7% of the estimated circulating blood volume; however, this does not account for the blood accumulation within the mediastinal tissue [18,19]. Postmortem SNAP 4Dx Plus testing was negative for *Dirofilaria immitis* antigen and antibodies to *Borrelia burgdorferi*, *Anaplasma phagocytophilum*/*A. platys*, and *Ehrlichia canis*/*E. ewingii*. It was concluded that the case was most consistent with spontaneous idiopathic thymic hemorrhage.

## 4. Discussion

This report describes the findings in a dog with presumed spontaneous idiopathic thymic hemorrhage, documented by PMCT and confirmed at autopsy. This case aligns with previously described features of the sentinel clot sign. In the present case, the hyperattenuating clot material measured 57 HU, consistent with values reported in the literature [1]. Accordingly, the PMCT findings support a sentinel clot-like structure of a focal blood clot surrounded by hemorrhage. However, the primary location of the hyperattenuating, clotted material within the pleural space differs from reports describing its typical localization adjacent to the source of hemorrhage [1,2]. This differing location from usual sentinel clots may indicate extension of mediastinal hemorrhage associated with thymic involution into the pleural space, which then accumulated within the pleural space and clotted, with the greater volume appreciated in the right pleural space associated with right-lateral recumbency patient positioning [20]. 

Postmortem redistribution and clot formation may also complicate interpretation of PMCT findings, given the location of the present case’s clot, despite its sentinel clot-like appearance. Factors such as the time between death and diagnostics, gravity, and patient positioning may cause the redistribution of blood [21]. Specifically, in humans, livor mortis typically develops within 2 h after death, when blood will pool within the vessels at gravity-dependent locations. However, in livor mortis, blood primarily remains within vessels, whereas in hemorrhage, blood can escape the vessels into surrounding spaces, consistent with the present case’s clot formation [22]. PMCT changes such as sedimentation are characterized by dependent layering of hyperattenuating cellular and iron-containing components beneath less attenuating serum [23,24], while postmortem clotting may occur in association with sedimentation and can demonstrate internal gravitational layering [23], which was not observed in this case. While postmortem changes such as plasma separation, hypostasis, and gravitational separation may increase the prominence of a pre-existing clot over time, these processes do not cause the formation of focal, hyperattenuating clots such as the clot observed in this case [25]. Rather, postmortem alterations may enhance visualization of underlying antemortem hemorrhage but are not considered primary causes of focal clot formation [25]. In this case, the non-sedimented, hyperattenuating soft tissue attenuating material on computed tomography, identified as a pleural clot formation on autopsy without evidence of trauma, supports an antemortem hemorrhagic process rather than a postmortem artifact. By comparison, “hammer heart” represents a circumferential, hyperattenuating cardiac clot and is considered an antemortem process related to residual cardiac contractility prior to death [24]. While “hammer heart” is specific to the pericardium, this circumferential pattern of hyperattenuating clot surrounded by lower-attenuation fluid is conceptually similar to a sentinel clot, which is typically focal and located adjacent to the site of a vascular or parenchymal injury [24]. These imaging characteristics support differentiation of the antemortem clot formation in this case from PMCT processes.

The age of the animal in the present case is within the reported normal documented time of thymic involution of 6–12 months [16], a process that is implicated in the development of spontaneous idiopathic thymic hemorrhage [12,13,26]. The PMCT similarly supports this diagnosis, with soft-tissue attenuating material in the pleural and mediastinal spaces, consistent with fluid and clotted blood [15]. In contrast, there was no definitive evidence of thymic pathology on PMCT or autopsy, such as masses within or adjacent to the thymus, despite the thymus being expanded with hemorrhage [15]. Autopsy and histology findings corresponded closely to previously documented lesions associated with spontaneous idiopathic thymic hemorrhage, including generalized tissue pallor (anemia), thymic hemorrhage and cranial hemomediastinum, as well as hemothorax, and lack of evidence of petechiae or hemorrhage elsewhere in the body [8]. 

Multiple causes of hemomediastinum and hemothorax can be excluded from this case. The absence of fractures, abrasions, lacerations, bruises, punctures, or sharp force injuries on either PMCT or autopsy makes traumatic injury an unlikely cause of the hemomediastinum and hemothorax [6]. The exclusion of blunt force trauma in this case was supported using PMCT, autopsy, and histopathology, rather than a single modality. Current forensic literature emphasizes integrating multiple diagnostic modalities to confirm evidence of traumatic injury, including autopsy, postmortem imaging, and ancillary testing [27,28]. In cases involving blunt force trauma, lacerations that are characterized by bridging and ragged wound edges are prominent [29]. In contrast, sharp force trauma typically produces wounds with linear or angular margins [30]. Rib fractures are among the most common injuries associated with thoracic trauma [31]. This case showed no evidence of rib fractures or lacerations, furthering the claim that blunt force trauma was an unlikely cause for the patient’s condition and death. While traumatic injury was considered unlikely, anticoagulant rodenticide toxicosis remained an important differential diagnosis. Anticoagulant rodenticides induce a hypocoagulable state by inhibiting vitamin K1-dependent clotting factors, resulting in spontaneous hemorrhage [32]. While the pleural space is the most common site of hemorrhage from anticoagulant rodenticides [7,32], other anatomic sites are frequently involved [7], including the skin, lungs, gastrointestinal tract, and peritoneal cavity [32]. Thoracic hemorrhage can be consistent with anticoagulant rodenticide toxicosis [7,32] and is more commonly associated with diffuse or multifocal bleeding rather than discrete focal clots, such as the pleural space clots observed in the present case [7,32,33]. However, without antemortem hematologic data in the current case, which includes coagulation parameters (prothrombin time and activated partial thromboplastin time), it is not possible to definitively exclude coagulopathy as a differential diagnosis [5,7,32].

Beyond facilitating differential exclusion in the present case, PMCT offers advantages over radiography in evaluating mediastinal disease. While radiography can provide information about masses and pleural effusion, confirmation and differentiation of these conditions often require advanced imaging, such as computed tomography [34]. Previous studies have emphasized the superiority of computed tomography over radiography due to its higher contrast resolution and lack of superimposition of internal structures [33]. Radiographic criteria, such as a distance greater than 6 cm between the tracheal bifurcation and thoracic midline, have been associated with cranial mediastinal masses and may warrant additional imaging, such as computed tomography [34]. Although radiology has a high specificity (98%) for confirming the absence of a mediastinal mass, its sensitivity for confirming the presence of a mass is limited (50%), further emphasizing the need for more advanced imaging techniques [34]. Given radiography’s limited ability to reliably differentiate mediastinal masses from pleural effusion [34], PMCT is an important diagnostic tool in cases such as the present, particularly for characterizing clot formation and surrounding fluid (hemorrhage). Although radiography was not used to establish the final diagnosis in this case, identification of the pleural clot formation and associated findings of spontaneous idiopathic thymic hemorrhage highlight the diagnostic value of PMCT.

The present case underscores the importance of considering spontaneous conditions when evaluating patients with clinical and pathologic findings that may initially suggest blunt force trauma. Although thoracic hemorrhage may raise concern for traumatic injury, spontaneous idiopathic thymic hemorrhage should be included among the differential diagnoses for hemomediastinum and hemothorax, specifically in young dogs. The use of a thorough clinical history, physical examination, diagnostics, and autopsy or histopathologic correlation is necessary to ensure an objective interpretation of the findings. In the present case, this principle was illustrated by the interpretation that all loose teeth were attributed to normal age-related loss of deciduous teeth. As emphasized in veterinary forensic pathology, a veterinary pathologist’s role in the investigation is to document and interpret findings independently, objectively, and systematically, without allowing the history of a case to influence the conclusions [35].

Limitations of this report include the use of a single animal, which limits the generalizability of the findings, and the lack of antemortem coagulopathy testing, which precluded the definitive assessment of coagulation abnormalities prior to death. Additionally, freezing and thawing of the body may have influenced the PMCT findings. Previous studies in humans and animals have demonstrated that freezing can alter CT attenuation values in a tissue-dependent manner [36]. For example, decreased attenuation has been reported in the vitreous humor and muscle following freezing, whereas increased attenuation has been observed in fat [36]. Therefore, the attenuation values measured during PMCT may not accurately reflect the tissues’ original state prior to death. The potential effects of freezing and thawing on the appearance and attenuation of the intrathoracic blood clots are unknown and should be considered when interpreting these findings. 

## 5. Conclusions

In this case, PMCT and the postmortem evaluation provided useful information for evaluating thoracic hemorrhage and considering possible alternative causes, such as traumatic injuries or coagulopathies, as well as presumptively differentiating antemortem and postmortem clot formation, providing useful information for future forensic case evaluation. The ability to presumably identify and characterize hemorrhage is useful in suspected animal abuse cases, particularly when determining whether trauma was responsible for the bleeding finding. Additional studies utilizing PMCT, autopsy, and antemortem hematologic data are needed to further validate the imaging characteristics of antemortem clot formation in veterinary pathology settings.

## Data Availability

The raw data supporting the conclusions of this article will be made available by the authors on request.

## Abbreviations

The following abbreviations are used in this manuscript:

PMCT	Postmortem Computed Tomography
HU	Hounsfield Units
